# Emergence of Bluetongue Virus Serotype 3 in Portugal (2024)

**DOI:** 10.3390/v16121845

**Published:** 2024-11-28

**Authors:** Sílvia C. Barros, Ana Margarida Henriques, Fernanda Ramos, Tiago Luís, Teresa Fagulha, André Magalhães, Inês Caetano, Fábio Abade dos Santos, Filipa O. Correia, Carlos C. Santana, Ana Duarte, Ruben Villalba, Margarida D. Duarte

**Affiliations:** 1 Virology Laboratory, Nacional Institute of Agrarian and Veterinarian Research, Quinta Do Marquês, Av. da República, 2780-157 Oeiras, Portugal; silvia.santosbarros@iniav.pt (S.C.B.); margarida.henriques@iniav.pt (A.M.H.); fernanda.ramos@iniav.pt (F.R.); tiago.luis@iniav.pt (T.L.); teresa.fagulha@iniav.pt (T.F.); andre.magalhaes@iniav.pt (A.M.); ines.caetano@iniav.pt (I.C.); fabio.abade@iniav.pt (F.A.d.S.); ana.duarte@iniav.pt (A.D.); 2 Faculdade de Ciências e Tecnologia, Universidade Nova de Lisboa, 2829-516 Caparica, Portugal; 3 Associate Laboratory for Animal and Veterinary Sciences (AL4AnimalS), Avenida da Universidade de Lisboa, 1300-477 Lisboa, Portugal; 4 CECAV-Centro de Ciência Animal e Veterinária, Faculdade de Medicina, Veterinária de Lisboa, Universidade Lusófona, Campo Grande 376, 1749-024 Lisboa, Portugal; 5 Clínica Veterinária VetHeavy, Serviços de Reprodução e Sanidade, Rua Diana de Liz, Parque do Iroma, 7006-801 Évora, Portugal; filipa.correia@vetheavy.pt; 6 VetBacelo, R. António Passaporte, 23, 7000 Évora, Portugal; vetbacelo.campo@gmail.com; 7 Faculdade de Medicina Veterinária, Centre for Interdisciplinary Research in Animal Health (CIISA), Universidade de Lisboa, Avenida da Universidade Técnica, 1300-477 Lisboa, Portugal; 8 European Union Reference Laboratory for Bluetongue, Laboratorio Central de Veterinaria, M-106 pk 1, 4, 28110 Madrid, Spain; rvillalba@mapa.es

**Keywords:** *Bluetongue virus*, BTV, BTV-3, serotype 3, emergence, sheep, Portugal

## Abstract

In September 2024, bluetongue virus serotype 3 (BTV-3) was first identified in Portugal, specifically in the Alentejo region (Évora District), using molecular diagnostic methods. The initial case involved a sheep exhibiting severe clinical symptoms, including head oedema, prostration, nasal discharge, and significant respiratory distress. A subsequent case was documented in another sheep from a different farm within the same district, which presented with high fever (41.5 °C), nasal discharge, and arthritis, ultimately resulting in mortality. Within one month of these initial detections, additional cases in both sheep and cattle were reported in neighbouring districts, indicating the virus rapid spread within the region. In response to this emerging threat, extensive monitoring efforts are being undertaken to delineate the distribution of BTV-3, and vaccination campaigns targeting this serotype have been initiated. These measures aim to mitigate the impact of the virus on livestock health and prevent further transmission.

## 1. Introduction

Bluetongue fever (BT) is a viral disease that affects domestic and wild ruminants [1,2] caused by bluetongue virus (BTV), a member of the genus *Orbivirus* in the family *Sedoreoviridae*. Although infections in other species, namely in carnivores, have been occasionally reported [1,3,4,5], the primary host is still ruminants. The disease is transmitted by blood-feeding insects of the genus *Culicoides*, which facilitate the transmission of the virus from infected animals in the viremia stage to healthy individuals [6,7], while some *Culicoides* species were found to support BTV multiplication after the ingestion of infected blood [8]. Therefore, in affected regions, BT exhibits a seasonal distribution, with increased incidence during the summer months, when rising temperatures promote the reproduction and persistence of *Culicoides* vectors in the environment.

BT is generally more severe in sheep, which typically exhibit high fever, depression, mucosal lesions in the mouth and nose, congestion of the coronary band, hypersalivation, nasal discharge, lameness, and mortality [2]. In contrast, cattle usually present with asymptomatic or mild clinical manifestations [9]. The severity of the disease in both species can vary significantly, as some serotypes exhibit higher virulence than others. Additionally, strains within the same serotype may also demonstrate differences in virulence. This is exemplified by the recently identified BTV3/NET2023 strain, which emerged in the Netherlands in 2023 and demonstrates unique pathogenic and spreading characteristics compared to the previous BTV-3 strain [10] associated with outbreaks in Italy between 2017 and 2022. After its emergence in the Netherlands in 2023 [9], this new BTV-3 strain has been well documented across Belgium (September 2023), Germany (October 2023), and Great Britain (November 2023) (WOAH disease reports). Notably, in the following summer, it was also detected in Denmark and France (July 2024); in Luxembourg, Norway, and Switzerland (August 2024); and in the Czech Republic (early September 2024) (WOAH disease reports).

In the context of the laboratorial diagnosis of BT, other viral diseases, such as contagious ecthyma, caused by the contagious ecthyma virus (CEV), a member of the genus *Parapoxvirus* within the subfamily *Chordopoxvirinae*, family *Poxviridae*, and malignant catarrhal fever (MCF), caused by ovine herpesvirus-2 (OvHV-2), which belongs to the *Gammaherpesvirinae* subfamily, *Herpesviridae* family, are frequently included as differential diagnoses.

In Portugal, the history of BTV has involved the emergence and management of various serotypes over the decades. Serotype 10 was the first identified in 1956, prompting significant concern, but it was successfully eradicated by 1960 through the implementation of live attenuated vaccination programmes. Serotype 4 emerged in 2004 and remains endemic, posing ongoing challenges for livestock health and management in the region. Serotype 2 was detected sporadically between 2004 and 2006, but it has not been observed in circulation since then, indicating a limited impact on local livestock [11]. Serotype 1 was first reported in 2007 (OIE reference: 6248/September/2007) and has since spread extensively across the country. To mitigate the risks associated with these serotypes, the vaccination of sheep against BTV-1 and BTV-4 has been mandatory in Portugal.

Here, we report the first detections of BTV-3 in mainland Portugal in September 2024.

## 2. Materials and Methods

### 2.1. Case Descriptions

The first case was an adult female crossbred sheep (Sheep 1) that died 4–5 days after sampling for laboratory investigation. The animal was part of a flock of 130 individuals originated from Évora District, which had been vaccinated against BTV-1 and BTV-4 in April 2024. Clinical signs were first observed on 1 September in 15 animals, including prostration, severe dyspnoea, wheezing, rhinorrhoea, and oedema of the head. At that time, blood samples from Sheep 1 were taken and submitted to the national reference laboratory on 12 September 2024 with a suspected diagnosis of BTV or epizootic haemorrhagic disease virus infection (EHDV).

The second case involved a male ovine (Sheep 2) also from Évora District, which developed fever (41.5 °C), rhinorrhoea, and arthritis prior to its death on September 7, with suspicion of contagious ecthyma (parapoxvirus) at the time. The onset of symptoms in the flock occurred on September 4, also affecting other sheep that died on the same day.

This herd encompassed around 3000 animals, of which, 150 died before 2 October 2024, representing a mortality rate of 5%.

### 2.2. Nucleic Acid Extraction

Lung, spleen, liver, and intestine samples from the dead sheep were homogenised at 20% (*w*/*v*) with phosphate-buffered saline by mechanical homogenisation with 0.5 mm zirconium beads (Sigma-Aldrich, St. Louis, MI, USA, EUA) using four cycles of 15 s at 3000 rpm with an interval of 10 s (Precellys^®^ Evolution, Paris, France) and then clarified at 3000 g for 5 min. Total nucleic acid extraction was carried out using 200 μL of the clarified supernatants, or using 200 μL blood samples using the IndiMag^®^ Pathogen Kit (Indical, Leipzig, Germany) in a KingFisher Flex extractor (ThermoFisher Scientific, Waltham, MA, USA), following the manufacturer’s protocol. Nucleic acids were preserved at −20 °C until use. Extractions were validated with an 18S rDNA qPCR [12] and an RT-qPCR for the detection of spiked synthetic RNA (VLP-RNA EXTRACTION, Meridian Life Science, Memphis, TN, USA) added to the sample (4 µL/sample) prior to extraction.

### 2.3. Molecular Investigations

Molecular investigations were conducted for BTV and its serotypes BTV-1, BTV-3, BTV-4, and BTV-8, as well as for epizootic haemorrhagic disease virus (EHDV), catarrhal malignant fever virus (CMFV), and contagious ecthyma virus (ORFV) as part of the differential diagnosis. The methodologies employed, as well as PCR kits used, are detailed in Table 1.

Amplifications were carried out in a Bio-Rad CFX96™ Thermal Cycler (Bio-Rad Laboratories Srl, Redmond, WA, USA) or T100 Thermal Cycler (Bio-Rad Laboratories Srl, Redmond, WA, USA) for real-time and conventional PCR, respectively. For all RT-qPCR methods, samples were classified as positive when a typical amplification curve was obtained and the cycle threshold (Ct) value was lower than or equal to a Ct value of 38 within 40 PCR cycles, and as negative when Ct > 38 or no Ct was obtained. For gel-based PCR, samples were considered as positive if the amplicon of the expected size was obtained.

**Table 1:** Viruses investigated in this study.

Viruses	Targeted Gene	Type of Method	PCR Kit Used	Method Reference
BTV	*ns3*	RT-qPCR	One-step NZYSpeedy RT-qPCR Probe kit, Nzytech, Lisbon, Portugal	[13]
BTV-1	*vp2*	RT-qPCR	One-step NZYSpeedy RT-qPCR Probe kit, Nzytech, Lisbon, Portugal	in-house method (see Table 2)
BTV-3	*vp2*	RT-qPCR	One-step NZYSpeedy RT-qPCR Probe kit, Nzytech, Lisbon, Portugal	[14]
BTV-4	*vp2*	RT-qPCR	One-step NZYSpeedy RT-qPCR Probe kit, Nzytech, Lisbon, Portugal	in-house method (see Table 2)
BTV-8	*vp2*	RT-qPCR	One-step NZYSpeedy RT-qPCR Probe kit, Nzytech, Lisbon, Portugal	[15] (see Table 2)
EHDV	*vp6*	RT-qPCR	One-step NZYSpeedy RT-qPCR Probe kit, Nzytech, Lisbon, Portugal	[16]
CMFV	ORF75	PCR	NZYTaq II 2x Green Master Mix, Nzytech, Lisbon, Portugal	[17]
ORFV	ORF045	PCR	NZYTaq II 2x Green Master Mix, Nzytech, Lisbon, Portugal	[18]

**Table 2:** Primer and probe sequences for in-house detection methods of BTV-1 and BTV-4.

Primer Forward (5′-3′)	Primer Reverse (5′-3′)	Probe FAM/BHQ-1 (5′-3′)	Serotype Detected
GAATGCATATGACATCAAGCAG	CGTCTTTCATCGTAACCCC	TGCYAYGTGGACGAGGGCATGC	BTV-1
GGTTAGAATGCCTGGACATG	GAGGCCACGGTCCGTGTC	TTCGGAAACGACGAACTGATGACG	BTV-4

### 2.4. Sanger Sequencing Analysis in the EURL

As an emerging disease in the country and following European Commission directives, a whole blood sample from Sheep 1 and an organ macerate from Sheep 2 were sent to the European Union Reference Laboratory (EURL-BT) in Spain. At the EURL, nucleic acid was extracted from 200 μL of EDTA blood or tissue homogenate using a BioSprint^®^ 96 DNA Blood Kit (Qiagen, Hilden, Germany) according to the manufacturer’s instructions. Nucleic acid was eluted in a final volume of 50 μL of nuclease-free water.

Two gel-based RT-PCR methods were performed. Primers were designed to amplify two distinct regions within the segment 2 sequence of BTV-3 with lengths of 501 and 630 base pairs. The primer sequences are as follows: primer 1 BTV-3 forward 5′-GGTATCCATCAGGCTCTCGC-3′; primer 1 BTV-3 reverse 5′-GCGCCTTAGTCGACACGATA-3′; primer 2 BTV-3 forward 5′-ACCGCGCAGGAATCTTGTTA-3′; primer 2 BTV-3 reverse 5′-GGGTTAGACCACCACACTCG-3′.

The PCR products were visualised in 2% horizontal electrophoresis agarose gel, purified using the QIAquick^®^ PCR Purification Kit (Qiagen), and directly sequenced using the ABI Prism BigDye Terminator v3.1 Cycle sequencing kit on a 3730 Genetic Analyser (Applied Biosystems, Foster City, CA, USA). Nucleotide sequences were assembled into consensus sequences using the DNA Sequencing Analysis Software Version 6.0 and SeqScape v.3.0 in Applied Biosystems.

## 3. Results

Both animals (Sheep 1 and Sheep 2) tested positive for BTV. Serotype-specific testing confirmed the presence of BTV-3, while both samples were negative for BTV-1, BTV-4, and BTV-8 (Table 3). Additionally, the blood sample from Sheep 1 tested negative for epizootic haemorrhagic disease virus (EHDV) (Table 3).

Sheep 2 tested negative for CEV but positive for OvHV-2 (Table 3).

The EURL laboratory confirmed the presence of the BTV-3 strain through serogroup- and serotype-specific RT-qPCR testing. Subsequently, amplicons from RT-qPCR-positive samples were generated using gel-based RT-PCR targeted to segment 2, sequenced by Sanger sequencing and submitted to GenBank (PQ609286 and PQ609287). Identical partial sequences were obtained from both samples, and these sequences were compared with homologous segments available in GenBank (Table 4).

## 4. Discussion

In mid-September 2024, BTV-3 was detected for the first time in Portugal, in sheep, specifically in the Évora District, marking a significant event, as at the time, no prior cases had been reported in the Iberian Peninsula. Shortly after, at the end of September 2024, Spain confirmed its first outbreak of BTV-3, occurring approximately two weeks after the initial detection in Portugal, which suggests that the virus is unlikely to have originated from the neighbouring country.

Partial sequences of segment 2 (PQ609286 and PQ609287) confirmed the presence of serotype 3. Although the partial sequences showed a 100% match to the BTV-3 strains circulating in northern Europe, the Netherlands (BTV3/NET2023, OR603993.1), and Germany (BTV3-BH44-23-GER, OZ119415.1), there is no documented history of animal imports from northern European countries affected by BTV-3. Furthermore, the observed differences in segment 2 products when compared to the BTV-3 strain BTV3/SAR 2018 from Sardinia, Italy, an isolate that has recently circulated in Europe, are substantial enough to exclude this strain as the source of the virus responsible for the emergence of BTV-3 in Portugal.

The epidemiological findings from the two cases fail to elucidate the origin and transmission dynamics of the infection. In the first farm, there was no importation of animals or introduction of new livestock into the herd. On the second farm, a batch of female lambs was introduced in mid-August 2024 from Azaruja (Évora), raising the possibility that asymptomatic carriers in the early stages of infection may have inadvertently introduced the virus. However, at this stage, any potential origin for the virus entrance such as the introduction of infected animals or the wind-driven spread of infected *Culicoides* remain purely speculative. Full sequencing of the BTV3 strain from Portugal is being conducted. Phylogenetic analyses will help identify how the Portuguese strain relates to other known BTV-3 strains and may provide insights into how the virus arrived in the region.

Interestingly, Sheep 2 was also positive for CMFV, a finding integral to the differential diagnosis process. CMFV is known to cause subclinical infections in sheep, which may reactivate during various disease processes. In this case, the reactivation of CMFV may have exacerbated the clinical severity associated with BTV-3 infection.

Approximately one month after this initial detection, several additional cases were reported in sheep and cattle, not only in the Évora District but also in neighbouring districts to the north (Castelo Branco), south (Beja), and west (Setúbal and Santarém). At that time, BTV-3 infection was confirmed on five cattle farms, with one case linked to clinical signs and increased neonatal mortality, a pattern rarely observed in other serotypes, except BTV-8. The increased virulence of the newly identified BTV-3, along with its apparent enhanced ability to infect carnivores [9] and its rapid geographical spread, raises significant concerns about its potential to cross species barriers and infect other hosts. In response, vaccination against BTV-3 with SYVAZUL BTV3 inactivated vaccine (Syva, Spain) is being implemented, reflecting the country’s commitment to controlling the disease and safeguarding its agricultural economy.

## Figures and Tables

**Table 3:** Results obtained in the molecular tests carried out in this investigation.

Case	Virus Investigated	Real-Time PCR Ct Value	Conventional PCR	Result
Sheep 1	BTV (pan RT-qPCR)	20.7	-	Positive
	BTV-1	N/A	-	Negative
	BTV-3	20.14	-	Positive
	BTV-4	N/A	-	Negative
	BTV-8	N/A	-	Negative
	EHDV	N/Ao	-	Negative
Sheep 2	BTV (pan RT-qPCR)	25.32	-	Positive
	BTV-1	N/A	-	Negative
	BTV-3	22.59	-	Positive
	BTV-4	N/A	-	Negative
	BTV-8	N/A	-	Negative
	CEV	-	No amplification	Negative
	OvHV-2	-	283 bp	Positive

**Table 4:** Homology comparison with other BTV segment 2 products deposited in GenBank.

			Top Similarity Matches in BLAST Analyses (October 2024)		
PCR Product (Viral Segment) Material ID at EURL AN	Length (bp)	nt Position in BTV-3/NET2023 (OR603993.1)	%	Strain	AN
Product 1 (partial seg-2) BTV3/3234/PT2024/blood(1) PQ609286	501	580–1080	100	BTV-3/NET2023	OR603993.1
			100	BTV3-BH44-23-GER	OZ119415.1
			98.41	BTV3-ZIM/2007	AJ585179.1
			98.01	BTV3-ZAF/2017/VR33	MG255620.1
			98.01	BTV3-ZAF/2017/VR11	MG255540.1
			97.81	BTV3-ZAF/2016/VR22	MT028400.1
			97.21	BTV3-TUN/2016	KY432370.1
			97.21	BTV3-SAR/2018	MK348538.1
Product 2 (partial seg-2) BTV3/3234/PT2024/blood(2) PQ609287	630	1846–2475	100	BTV-3/NET2023	OR603993.1
			100	BTV3-BH44-23-GER	OZ119415.1
			98.41	BTV3-ZIM/2007	AJ585179.1
			97.21	BTV3-TUN/2016	KY432370.1
			97.21	BTV3-SAR/2018	MK348538.1

AN—GenBank accession numbers.

## Data Availability

The data supporting the results of this study can be obtained by contacting the authors; however, farm identities are not disclosed due to confidentiality and the right to privacy of the property owners. The nucleotide sequences have been submitted to GenBank under the following accession numbers: PQ609286 and PQ609287.
